# Comparative analysis of the lumboperitoneal shunt versus ventriculoperitoneal shunt for leptomeningeal metastasis-associated hydrocephalus in non-small cell lung cancer

**DOI:** 10.1007/s00701-025-06490-y

**Published:** 2025-03-19

**Authors:** Minjoon Kim, Chaejin Lee, Sang-Youl Yoon, Seong-Hyun Park, Jeong-Hyun Hwang, Kyunghun Kang, Eunhee Park, Sunha Choi, Shin Yup Lee, Seung Soo Yoo, Yee Soo Chae, Ki-Su Park

**Affiliations:** 1 Department of Neurosurgery, School of Medicine, Kyungpook National University, 101 Dongin-dong 2 Ga, Jung-gu, Daegu, 700-422 Republic of Korea; 2 Department of Neurology, School of Medicine, Kyungpook National University, Daegu, Korea; 3 Department of Rehabilitation Medicine, School of Medicine, Kyungpook National University, Daegu, Korea; 4 Division of Pulmonology, Department of Internal Medicine, School of Medicine, Kyungpook National University, Daegu, Korea; 5 Department of Hemato/Oncology, School of Medicine, Kyungpook National University, Daegu, Korea

**Keywords:** Non-small-cell lung, Carcinoma, Meningeal carcinomatosis, Hydrocephalus, Ventriculoperitoneal shunt

## Abstract

**Purpose:**

Leptomeningeal metastasis (LM)-associated hydrocephalus is a rare but severe complication of non-small cell lung cancer (NSCLC). The spread of malignant cells to the leptomeninges obstructs cerebrospinal fluid flow and increases intracranial pressure (ICP). This study compared the outcomes of lumboperitoneal (LP) and ventriculoperitoneal (VP) shunt surgeries in managing LM-associated hydrocephalus, focusing on symptom-free periods (SFPs) and overall survival (OS).

**Methods:**

A retrospective analysis was conducted on 43 NSCLC patients with LM-associated hydrocephalus who underwent shunt surgery between 2017 and 2024. Patients were classified into LP (*n* = 23) and VP (*n* = 20) groups. Clinical characteristics, surgical outcomes, and survival rates were analyzed using Kaplan–Meier survival and Cox regression analyses. Karnofsky performance status (KPS), increased intracranial pressure (IICP) symptoms, and postoperative epidermal growth factor receptor-tyrosine kinase inhibitor (EGFR-TKI) treatment were evaluated for prognostic significance.

**Results:**

No significant difference was observed between VP and LP shunt surgeries regarding SFP (VP: 5.02 ± 1.29 months, LP: 7.50 ± 2.86 months, *p* = 0.906) or OS (VP: 8.43 ± 1.89 months, LP: 9.62 ± 3.20 months, *p* = 0.820). High preoperative KPS, absence of ICP symptoms, and postoperative EGFR-TKI treatment were significantly associated with improved SFP and OS (*p* < 0.05). LP shunt surgery had shorter anesthesia and fewer complications compared to VP shunt surgery, representing a viable option for patients unsuitable for general anesthesia.

**Conclusion:**

LP and VP shunt surgeries are equally effective for patients with LM-associated hydrocephalus in NSCLC. LP shunt surgery under local anesthesia could be recommended for its safety and patient acceptability. Favorable prognostic factors, including high KPS, absence of ICP, and postoperative EGFR-TKI treatment, should guide individualized treatment strategies to enhance patient outcomes and quality of life.

## Introduction

Leptomeningeal metastasis (LM) is caused by the spread of malignant cancer cells to the leptomeninges, the two innermost layers of the meninges surrounding the brain and spinal cord. This rare and aggressive condition is commonly associated with advanced cancer stages [5, 21]. The most common primary tumor responsible for LM is lung cancer, particularly non-small cell lung cancer (NSCLC), followed by breast cancer and melanoma [5, 6, 21, 26]. Clinical symptoms of LM include neurological deficits, cognitive impairments, and cranial nerve dysfunction, with diagnosis typically confirmed through cerebrospinal fluid (CSF) analysis and magnetic resonance imaging (MRI) [3, 12, 27, 32]. LM is increasingly encountered in clinical practice nowadays, largely due to advancements in MRI-based diagnostics and cancer treatments that have significantly extended patient survival [21]. As the patient’s lifespan becomes longer, they become susceptible to developing late-stage complications such as LM. However, despite notable progress in oncologic therapies, the prognosis for LM remains poor, with median survival of 2–4 months despite therapy and 4–6 weeks if untreated [32].

LM-associated hydrocephalus is a critical complication of LM, which occurs due to the obstruction of the CSF flow within the ventricular system by cancer cells. Based on the available literature, about 50%–70% of LM patients experience altered CSF dynamics, but only 1%–5% develop symptomatic hydrocephalus, manifesting as either communicating or non-communicating type depending on the CSF flow dynamics and the location of metastatic deposits [26]. Despite the relatively low prevalence and survival rates of LM-associated hydrocephalus, treatment is needed due to neurological symptoms such as severe headaches and decreased consciousness, often caused by a rapid increase in intracranial pressure (ICP) [7, 10, 21, 22]. Treatment interventions for LM-associated hydrocephalus vary depending on the patient’s condition and hydrocephalus subtype. Ventricular shunting surgery and endoscopic third ventriculostomy (ETV) are commonly performed and are effective in lowering ICP by diverting CSF in the ventricles. However, lumbar drainage and palliative care focused solely on pain management without surgery may be considered in cases when surgical intervention is not feasible due to poor patient conditions [7, 10, 22].

Lumboperitoneal (LP) shunt surgery has been a recently emerging viable alternative, particularly for patients with LM-associated communicating hydrocephalus. LP shunt surgery offers several potential advantages, including a less invasive approach, a lower risk of complications associated with inserting a ventricular catheter through the brain, and the possibility of using local anesthesia or minimal sedation, thereby avoiding the need for general anesthesia [35, 38]. However, comparative studies of VP and LP shunt surgeries are limited despite these potential benefits. Therefore, conducting such research is critical to establishing evidence-based guidelines that could optimize treatment outcomes for LM-associated communicating hydrocephalus, particularly as LP shunts may provide a safer, more effective option for certain patient populations [10, 38].

This study aimed to compare the effectiveness of LP and VP shunt surgeries in managing LM-associated hydrocephalus. Specifically, we focused on analyzing the outcomes of these two surgical interventions in patients with LM-associated hydrocephalus due to NSCLC, providing a more objective comparison and offering deeper clinical insights into the management of this challenging condition.

## Material and methods

### Patient selection and data acquisition

The Institutional Review Board of Kyungpook National University Chilgok Hospital approved this study (KNUCH 2025–01–038). We conducted a retrospective comparative analysis of LP and VP shunt surgeries for communicating hydrocephalus of LM from January 2017 to July 2024. Medical records, imaging studies, histopathological findings, LM presentation, possible predisposing factors, treatment, and outcomes were retrospectively reviewed.

The inclusion criteria for patients with LM-related hydrocephalus for this study were (1) non-small cell lung cancer; (2) confirmed diagnosis of LM according to the joint European Association of Neuro-Oncology (EANO)—European Society for Medical Oncology (ESMO) guideline [15]; (3) clinical or radiological evidence of hydrocephalus, with clinical signs such as headaches, nausea, vomiting, cognitive decline, gait disturbances, or other neurological deficits due to increased ICP and radiological signs such as enlarged ventricles on brain imaging; (4) adults aged ≥ 18 years (to focus on adult-onset LM); (5) patients eligible for surgical treatment as judged by respiratory and hematological oncology specialists (S.H.C., S.S.Y., S.Y.L., and E.S.C.) based on clinical and imaging findings.

The exclusion criteria for patients with LM-related hydrocephalus in this study were as follows: (1) neurological impairment clearly caused by conditions other than hydrocephalus, such as brain metastases or stroke; (2) obstructive hydrocephalus, which precludes the option of LP shunt surgery; and (3) a follow-up period of less than three months.

### Leptomeningeal metastasis

The joint 2023 guidelines from the EANO and the ESMO recommend confirming and classifying leptomeningeal metastases based on the following criteria [11]: (1) interpretation of gadolinium-enhanced brain MRI, (2) cytology or meningeal biopsy, and (3) typical clinical signs. MRI findings are classified into (A) linear leptomeningeal enhancement, (B) nodular leptomeningeal enhancement, (C) combined linear and nodular enhancement, and (D) no evidence of enhancement, with or without hydrocephalus.

Type 1 is “confirmed LM,” indicating cytology or biopsy-positive cases. Type 2 includes cases with negative or non-performed cytology or biopsy. These cannot be classified as “confirmed.” However, MRI-positive cases (types A, B, and C) can be diagnosed as “probable LM.” If a brain MRI does not show leptomeningeal enhancement, a patient with typical clinical signs can only be diagnosed with “possible LM.”

This study enrolled only patients with confirmed hydrocephalus and typical clinical signs, allowing for classification into 8 subtypes from 1A to 2D. Table 1 summarizes the patients’ diagnostic classifications.

**Table 1:** Clinical characteristics of patients with LM-associated hydrocephalus in NSCLC

Variables	All patients (*n* = 43)	VP shunt (*n* = 20)	LP shunt (*n* = 23)	P-value
Age in years at shunt operation				
Average (range)	63.51 ± 10.12	62.65 ± 11.075	64.26 ± 9.39	0.608
Gender (%)				
Male	23 (53.5)	9 (45)	14 (60.9)	0.298
Female	20 (46.5)	11 (55)	9 (39.1)	
Underlying disease (%)				
HTN	17 (39.5)	5 (25.0)	12 (52.2)	0.069
DM	6 (14.0)	2 (10.0)	4 (17.4)	0.669
Hyperlipidemia	11 (25.6)	3 (15.0)	8 (34.8)	0.175
CVA	5 (11.6)	2 (10.0)	3 (13.0)	0.463
Anticoagulant use (%)	15 (34.9)	5 (25)	10 (43.5)	0.205
EANO-ESMO subtype (%)				
1A	20 (46.5)	11 (55)	9 (39.1)	0.246
1B	2 (4.7)	0 (0)	2 (8.7)	
1C	4 (9.3)	1 (5)	3 (13.0)	
1D	3 (7.0)	3 (15)	0 (0)	
2A	6 (14.0)	2 (10)	4 (17.4)	
2B	6 (14.0)	2 (10)	4 (17.4)	
2C	1 (2.3)	0 ()	1 (4.3)	
2D	1 (2.3)	1 (5)	0 (0)	
CSF cytology ( +) (%)	29 (67.4)	15 (75)	14 (60.1)	0.142
Symptom (%)				
IICP	34 (79.4)	18 (90)	16 (69.6)	0.324
NPH symptom only	9 (20.9)	2 (10)	7 (30.4)	
Extracranial cancer control (%)				
Good	30 (69.8)	13 (65)	17 (73.9)	0.526
Poor	13 (30.2)	7 (35)	6 (26.1)	
KPS (%)				
Low (0–40)	25 (58.1)	14 (70)	11 (47.8)	0.142
High (50–100)	18 (41.9)	6 (30)	12 (52.2)	
Genetic mutation (%)				
EGFR mutation	31 (72.1)	15 (75)	16 (69.6)	0.692
PD-1/PD-L1 ( +)	16 (37.2)	7 (35)	9 (39.1)	0.780
ALK rearrangement	1 (2.3)	0 (0)	1 (4.3)	1.000
ROS1 rearrangement	2 (4.6)	0 (0)	2 (8.7)	0.491
TKI therapy after LM (%)	16 (37.2)	6 (30)	10 (43.5)	0.362
Intrathecal chemotherapy (%)	6 (14.0)	4 (20)	2 (8.7)	0.393
Symptom-free period (months)	6.25 ± 1.57	5.02 ± 1.29	7.50 ± 2.86	0.906
Overall survival (months)	8.72 ± 1.75	8.43 ± 1.89	9.62 ± 3.20	0.820

*VP* ventriculoperitoneal; *LP* lumboperitoneal; *LM* leptomeningeal metastasis; *NSCLC* non-small cell lung cancer; *HTN* hypertension; *DM* diabetes mellitus; *CVA* cerebrovascular accident; *CSF* cerebrospinal fluid; *EANO* European Association of Neuro-Oncology; *ESMO* European Society for Medical Oncology; *IICP* increased intracranial pressure; *KPS* Karnofsky Performance Scale; *EGFR* epidermal growth factor receptor; *ALK* anaplastic lymphoma kinase; *ROS1* c-ros oncogene 1; *TKI* tyrosine kinase inhibitors; *PD-1/PD-L1* programmed death-1 and programmed death-ligand 1

Values are presented as mean ± standard deviation (SD)

^*^P-values < 0.05 were considered statistically significant

### Ventriculoperitoneal shunt surgery

The patient was placed in a supine position under general anesthesia with lidocaine, propofol, and rocuronium and maintenance with desflurane. The head was shaved and sterilized, with particular attention to the entry point for Kocher’s point, which is located approximately 2–3 cm lateral to the midline and 1 cm anterior to the coronal suture on the right side of the head. A small incision was made over the Kocher’s point, followed by the creation of a burr hole in the skull. The dura mater was carefully exposed and opened using a small incision. A ventricular catheter was introduced through the burr hole and directed into the lateral ventricle, using anatomical landmarks and, if available, image guidance to ensure its correct positioning within the frontal horn of the lateral ventricle. Once the catheter was appropriately placed, the CSF flow was confirmed by spontaneous fluid outflow through the catheter. A subcutaneous tunnel was created from the head to the abdomen, usually along the chest wall, where the peritoneal catheter would be passed. The distal catheter was introduced into the peritoneal cavity through a small abdominal incision, ensuring proper positioning for absorption of the diverted CSF. The ventricular catheter was connected to the Certas™ Plus valve (Codman, Raynham, Massachusetts), which regulates the CSF flow, and the peritoneal catheter. The entire shunt system was carefully checked for proper function. The surgical incisions on the scalp and abdomen were closed in layers with sutures.

### Lumboperitoneal shunt surgery

The patient was positioned in a left lateral decubitus posture under mild sedation (a combination of propofol and midazolam) to ensure the patient’s comfort while maintaining a responsive level of consciousness. A dermal marking for the entry point was made on the patient’s back 1 cm lateral to the midline of the spinous process at the level of the inferior border of the L3 vertebral body and in the paraumbilical region for the abdominal incision. The Tuohy needle was inserted at the entry point on the back and advanced toward the spinal canal, targeting the L2–3 level under fluoroscopic guidance. When the Tuohy needle reached the CSF space, the spinal catheter was inserted. A strata adjustable programmable valve (Medtronic Neurologic Technologies, Medtronic Inc., Goleta, CA, USA) was connected to the spinal catheter. Afterward, the distal catheter was connected to the valve and inserted into the abdominal cavity in a conventional manner. The entire shunt system was carefully checked for proper function. The surgical incisions on the scalp and abdomen were closed in layers with sutures.

### Clinical assessment

Surgical characteristics, including type of anesthesia, symptom improvement, procedure duration, and postoperative complications, were compared between the VP and LP shunt surgery groups. Given the short life expectancy, clinical symptoms could deteriorate within days despite hydrocephalus-related symptom relief following CSF shunting. Therefore, "symptom improvement" was defined as early improvement in consciousness for patients with decreased consciousness or as gait and cognitive function improvement for those with NPH symptoms.

Survival analysis was conducted to compare overall survival (OS) and the symptom-free period (SFP) between the VP and LP shunt surgery groups. SFP was defined as the recurrence of preoperative symptoms despite shunt surgery and valve pressure adjustments. Multivariate analysis included covariates such as KPS, cancer control status, increased ICP symptoms, intrathecal chemotherapy, and EGFR-TKI treatment. Patients with a KPS score of ≤ 40 were classified as having "low KPS," while those with a score of ≥ 50 were categorized as having "high KPS." Cancer control was considered "good" in patients with stable or responding disease under maintenance chemotherapy and "poor" in those with progressive disease.

Additionally, given the survival benefits of targeted chemotherapy, we reviewed patients’ genetic profiles and treatment regimens. However, postoperative EGFR-TKI treatment was the only analyzable factor due to the limited number of patients with ALK (one case) and ROS1 (two cases) rearrangements. Although PD-1/PD-L1 mutations were present in 16 patients (37.2%), none received immune checkpoint inhibitor therapy after shunt surgery.

### Statistical analysis

Various statistical tests (including T-tests, mean comparisons, association analyses, Chi-square tests, and proportion tests) were applied as appropriate. Kaplan–Meier survival and Cox regression multivariate analyses were utilized to evaluate the SFP and OS following shunt surgery. Additionally, SFP was defined as the point at which symptoms returned to the preoperative level despite shunt surgery and appropriate valve pressure adjustment. A *p* < 0.05 indicated statistical significance. All analyses were conducted using SPSS Statistics for Windows, Version 19.0 (SPSS Inc., Chicago, IL), to examine the relationships among variables.

## Results

### Clinical characteristics of patients with leptomeningeal metastasis-associated hydrocephalus in non-small cell lung cancer

A total of 46 patients met the inclusion criteria; however, three were excluded, leaving 43 patients in the final analysis. Two patients were excluded due to cerebellar masses causing obstructive hydrocephalus, and one due to a follow-up period of only one month. Table 1 presents the clinical characteristics of the patients with LM-associated hydrocephalus and NSCLC. Among the 43 enrolled patients, 20 patients (46.5%) underwent VP shunt surgery, while 23 patients (53.5%) underwent LP shunt surgery. Most were diagnosed by the EANO-ESMO guidelines and CSF cytology. However, there was no statistical significance in age, sex, underlying disease, preoperative anticoagulant use, LM subclassification, CSF cytology, preoperative KPS and cancer control state, or symptoms among surgical methods.

### Surgical characteristics between lumboperitoneal and ventriculoperitoneal shunt surgeries

Table 2 presents the characteristics of surgery between the two groups. From an anesthetic perspective, 20 patients (46.5%) who underwent VP shunt surgery received general anesthesia, while 23 patients (53.5%) who underwent LP shunt surgery received local anesthesia (*p* < 0.001). The total anesthesia time was significantly longer in VP shunt surgery at 108.1 ± 24.8 min compared to 67.3 ± 14.5 min in LP shunt surgery (*p* < 0.001). However, the pure procedure times were 63.8 ± 21.9 min and 56.3 ± 14.2 min, respectively, without statistically significant difference (*p* = 0.312).

**Table 2:** Surgical characteristics between lumboperitoneal and ventriculoperitoneal shunt procedures

Variables	All patients (*n* = 43)	VP shunt (*n* = 20)	LP shunt (*n* = 23)	*P*-value
Anesthetic type (%)				
General anesthesia	20 (46.5)	20 (100)	0	< 0.001
Mild sedation	23 (53.5)	0	23 (100)	
Anesthesia time (minutes)	79.7 ± 32.3	108.1 ± 24.8	67.3 ± 14.5	< 0.001
Procedure time (minutes)	59.7 ± 18.4	63.8 ± 21.9	56.3 ± 14.2	0.312
Postoperative symptom improvement (%)	35 (81.4)	16 (80.0)	16 (69.6)	0.726
Postoperative complication (%)	6 (14.0)	2 (10.0)	4 (17.3)	0.428
CSDH	2 (4.7)	1 (5.0)	1 (4.3)	1.00
Wound dehiscence	2 (4.7)	0 (0.0)	2 (8.6)	0.492
Catheter dislodgement	2 (4.7)	1 (5.0)	1 (4.3)	1.00
Brain hemorrhage around the catheter (%)	2 ( 4.7)	2 (10.0)	0	< 0.001

*VP* ventriculoperitoneal; *LP* lumboperitoneal; *LM* leptomeningeal metastasis; *CSDH* chronic subdural hematoma; *IICP* increased intracranial pressure

Values are presented as mean ± standard deviation (SD)

^*^ *P*-values of < 0.05 were considered statistically significant

Overall, postoperative symptom improvement was observed in 35 out of 43 patients (81.4%). Specifically, 16 out of 20 patients (80.0%) who underwent VP shunt surgery and 19 out of 23 patients (82.6%) who underwent LP shunt surgery showed improvement. However, the difference was not statistically significant (*p* = 1.000). Postoperative complications included chronic subdural hematoma in 2 patients, wound dehiscence in 2 patients, and catheter dislodgement in 2 patients. However, no correlation was found between the surgery type and these complications. Although not fatal, brain hemorrhage around the shunt catheter occurred in 2 patients (10.0%) following VP shunt surgery (*p* < 0.001). Additionally, no immediate postoperative deaths were noted in any of the patients.

### Univariate and multivariate analyses of prognostic determinants in LM-associated hydrocephalus

**Fig. 1:**
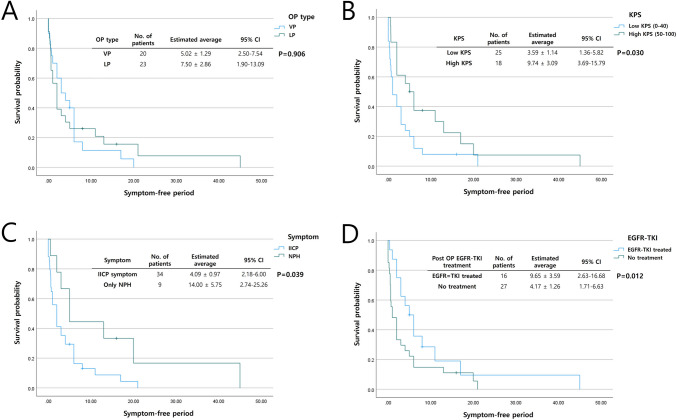
Kaplan–Meier survival curve showing symptom-free period after shunt surgery. (**A**) There was no statistically significant difference for symptom-free period between VP and LP groups. (**B**, **C**, **D**) In the high preoperative KPS group, patients who did not present with IICP symptoms and patients treated with EGFR-TKI postoperatively showed prolonged symptom-free period after shunt surgery, each with statistical significance (*p* < 0.05)

**Fig. 2:**
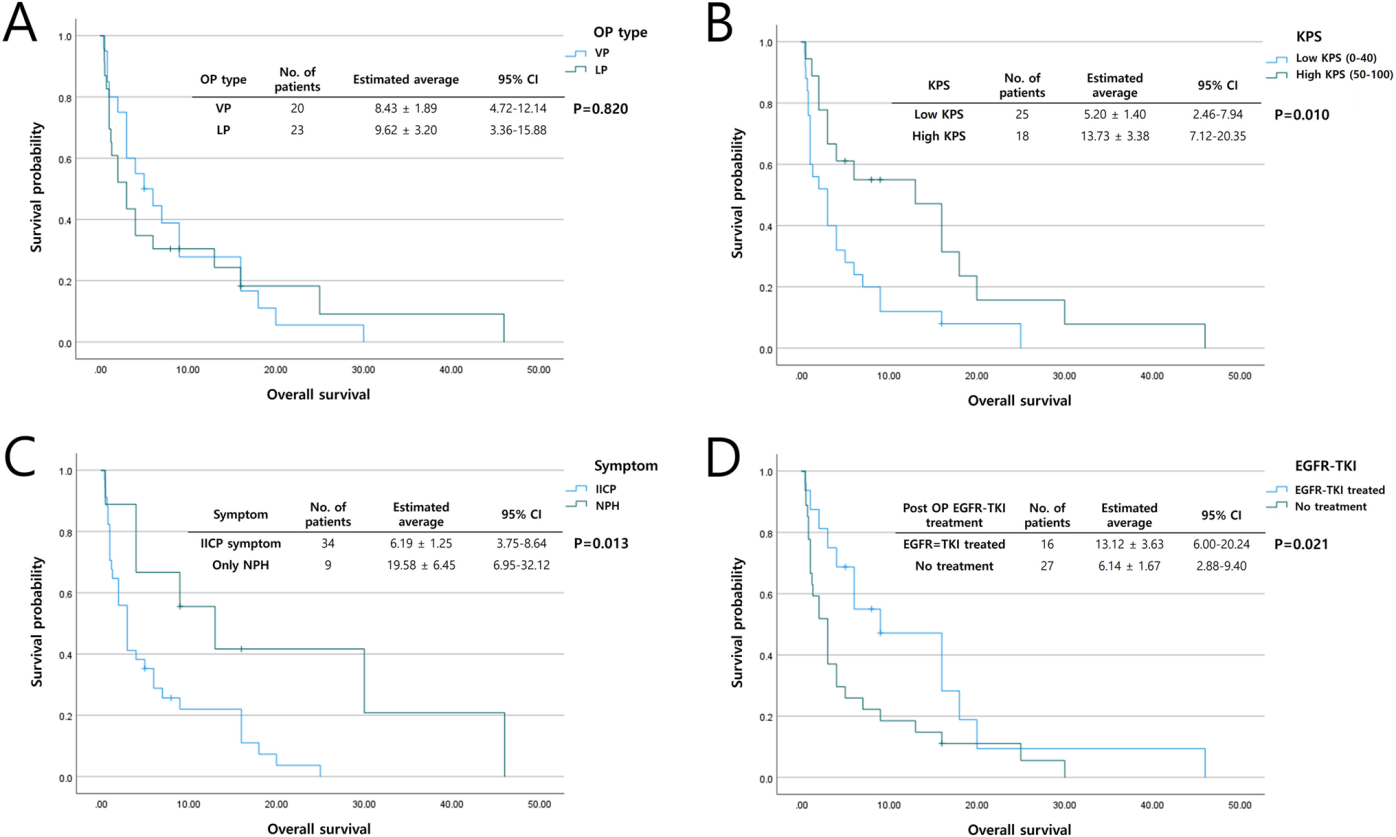
Kaplan–Meier survival curve showing overall survival after shunt surgery. (**A**) There was no statistically significant difference for overall survival between VP and LP groups. (**B**, **C**, **D**) In the high preoperative KPS group, patients who did not present with IICP symptoms and patients treated with EGFR-TKI postoperatively showed prolonged overall survival after shunt surgery, each with statistical significance (*p* < 0.05)

Figures 1 and 2 show SFP and OS according to the Kaplan–Meier survival analysis, respectively.

For the SFP, no statistical significance between shunt surgery types was identified (VP shunt surgery: 5.02 ± 1.29 months vs. LP shunt surgery 7.50 ± 2.86 months; *p* = 0.906). However, patients who did not present with IICP symptoms (only presenting with NPH symptoms: 14.00 ± 5.75 months vs. IICP: 4.09 ± 0.97 months; *p* = 0.039) and patients postoperatively treated by EGFR-TKI (postoperative EGFR-TKI treatment: 9.65 ± 3.59 months vs. no postoperative EGFR-TKI treatment: 1.26 ± 1.26 months; *p* = 0.012) showed prolonged SFP after shunt surgery, each with statistical significance, in the high preoperative KPS group (high KPS: 9.74 ± 3.09 months vs. low KPS: 3.59 ± 1.14 months; *p* = 0.030).

No statistical significance between shunt surgery types regarding OS was detected (VP shunt surgery: 8.43 ± 1.89 months vs. LP shunt surgery: 9.62 ± 3.20 months, *p* = 0.820). However, patients who did present with IICP symptoms (only presenting with NPH symptoms: 19.58 ± 6.45 months vs. IICP: 6.19 ± 1.25 months; *p* = 0.013) postoperatively and patients treated by EGFR-TKI (postoperative EGFR-TKI treatment: 13.12 ± 3.63 months vs. no postoperative EGFR-TKI treatment: 6.14 ± 1.67 months; *p* = 0.021) showed prolonged OS after shunt surgery, each with statistical significance, in the high preoperative KPS group (high KPS: 13.73 ± 3.38 months vs. low KPS: 5.20 ± 1.40 months; *p* = 0.010).

Table 3 shows the univariate and multivariate Cox regression analyses for SFP.

**Table 3:** Univariate and multivariate analyses for the symptom-free period of patients with leptomeningeal metastasis-associated hydrocephalus

	Univariate			Multivariate		
	Hazard ratio	95% CI	*p*-value	Hazard ratio	95% CI	*p*-value
OP type						
VP shunt surgery	0.96	0.51–1.83	0.964	-	-	
IT-MTX						
Not performed	2.91	0.89–9.58	0.078	2.91	0.89–9.58	0.078
Primary cancer control						
Poor	1.52	0.76–3.05	0.237	1.52	0.76–3.05	0.237
Preoperative KPS						
Low (< 50)	1.97	1.03–3.80	0.042	1.99	1.03–3.84	0.041
Symptom						
IICP	2.29	0.99–5.30	0.054	2.38	1.01–5.60	0.048
Postoperative TKI						
Not performed	1.88	0.94–3.69	0.065	1.89	0.96–3.71	0.065

*OP* operation; *VP* ventriculoperitoneal; *KPS* Karnofsky Performance Status; *IICP* increased intracranial pressure; *TKI* tyrosine kinase inhibitor

^*^ *P*-values of < 0.05 were considered statistically significant

In the univariate analysis, the preoperatively low KPS group showed a significant difference (hazard ratio [HR]: 1.97, 95% confidence interval [CI]: 1.03–3.80, *p* = 0.042). Furthermore, the presence of IICP symptoms before surgery showed a marginal significance (HR: 2.29, 95% CI: 0.99–5.30, *p* = 0.054).

In the multivariate analysis, the preoperatively low KPS (HR: 1.99, 95% CI: 1.03–3.84, *p* = 0.041) and the presence of IICP symptoms before surgery (HR: 2.38, 95% CI: 1.01–5.60, *p* = 0.048) were identified as significant covariates. Low KPS and the presence of IICP symptoms may be related to poor SFP.

 Table 4 shows the univariate and multivariate Cox regression analyses for OS.

**Table 4:** Univariate and multivariate analyses for the overall survival of patients with leptomeningeal metastasis-associated hydrocephalus

	Univariate			Multivariate		
	Hazard ratio	95% CI	*p*-value	Hazard ratio	95% CI	*p*-value
OP type						
VP	1.07	0.57–2.03	0.827	-	-	
IT-MTX						
Not performed	2.60	0.79–8.55	0.073	2.65	0.80–8.76	0.111
Primary cancer control						
Poor	1.91	0.94–3.88	0.074	1.91	0.94–3.88	0.074
Preoperative KPS						
Low (< 50)	2.32	1.17–4.62	0.016	2.37	1.19–4.72	0.014
Symptom						
IICP	3.04	1.17–7.93	0.023	3.33	1.26–8.82	0.015
Postoperative TKI						
Not performed	2.00	1.00–3.99	0.049	2.03	1.02–4.07	0.045

*VP* ventriculoperitoneal; *LP* lumboperitoneal; *IT-MTX* intrathecal methotrexate; *KPS* Karnofsky Performance Scale *IICP* increased intracranial pressure; *TKI* tyrosine kinase

^*^ *P*-values of < 0.05 were considered statistically significant

In the univariate analysis, the preoperatively low KPS group (HR: 2.32, 95% CI: 1.17–4.62, *p* = 0.016], the presence of IICP symptoms before surgery (HR: 3.04, 95% CI: 1.17–7.93, *p* = 0.023), and no postoperative EGFR-TKI treatment (HR: 2.00, 95% CI: 1.00–3.99, *p* = 0.049) showed statistically significant differences.

In the multivariate analysis, the preoperatively low KPS (HR: 2.37, 95% CI: 1.19–4.72, *p* = 0.014), the presence of IICP symptoms before surgery (HR: 3.33, 95% CI: 1.26–8.82, *p* = 0.015), and no postoperative EGFR-TKI treatment (HR: 2.03, 95% CI: 1.02–4.07, *p* = 0.045) showed statistically significant differences. Furthermore, the low KPS, the presence of IICP, and no postoperative EGFR-TKI treatment may be related to poor OS.

## Discussion

LM is characterized by an extremely poor prognosis without well-established consensus on its management. LM is inherently chemoresistant due to the presence of the blood–brain barrier, complicating drug delivery [2, 17, 25]. Additionally, the benefit and the methodology of the central nervous system (CNS) irradiation is largely controversial [18]. Despite the evolvement of intrathecal therapy and molecular targeted therapies with excellent CNS penetration and clinical outcomes [1, 2, 13, 24], a recent review on LM due to NSCLC reports a median survival of only 1–3 months with conventional therapy and 3–11 months with novel therapies [2]. Thus, the therapeutic goal for LM still remains palliative.

In LM-associated hydrocephalus (without surgical treatment), increased ICP can lead to sudden death or severe neurological deterioration. Even if immediate death is avoided, immobilized patients often face fatal medical complications. Lowering ICP through the CSF diversion procedure is crucial for patients with LM-associated hydrocephalus. Numerous previous studies reported that surgical treatment improves symptoms and survival [4, 8, 10, 14, 15, 19, 23, 31, 35]. In terms of OS, Jung et al. (2014) explored the prognostic significance of surgically treated hydrocephalus in LM patients, showing improved OS compared to non-surgically treated patients (median OS of 5.7 months vs. 1.7 months in untreated patients), although without statistically significant differences [8]. In our study, the rate of symptom improvement and median OS after shunt surgery were consistent with previous reports (Table 5) [10].

**Table 5:** Clinical characteristics of patients with palliative shunt surgery after leptomeningeal metastasis related hydrocephalus

Study	Primary pathology	No. of patients	Presenting symptoms	Intervention (*n*)	Symptom improvement, overall rate	Symptom improvement by symptom (*n*)	Complications overall %; type (*n*)	Perioperative mortality	Median survival from LMD diagnosis (months [IQR])	Median survival from surgery, [IQR]
This study	NSCLC (43)	43	Gait issues (27), Altered mentality (25), headache (19), cognitive changes (17), nausea and vomiting (13), urinary incontinence (9), cranial nerve palsy (6), visual disturbance (4), seizure (3)	LPS (23), VPS (20)	35/43 (81.3%)	N.R	14.0%; catheter dislodgement (2), subdural hematoma (2), wound dehiscence (2)	7/43 (16.3%)	6 months [2–14 months]	4 months [1–9 months]
Lokich et al. [20]	Lung adenocarcinoma (2), breast (1)	3	Gait issues (3), headache (2), nausea (1), cognitive changes (1)	VPS (3)	3/3 (100%)	Gait issues (3), headache (2), nausea (1), cognitive changes (1)	N.R	0/3 (0%)	N.R	6 months (4–7.5 months]
Omuro et al. [21]	Breast (23), lung (6), melanoma (3), others (5)	37	Headache (24), nausea/vomiting (18), cognitive changes (19), gait issues (20), spinal symptoms (10), hemiparesis (12), papilledema (4)	VPS (37/37)	27/37 (77%)	Most had decreased headache, nausea/vomiting, and improved level of alertness^1^	11%; shunt malfunction requiring revision (3), subdural hematoma (1), infection (0), peritoneal carcinomatosis (0)	0/37 (0%)	4 [3 days to > 3.6 years]^2^	2 months [2 days to > 3.6 years]b
Lee et al. [22]	NSCLC (29), SCLC (2), mixed NSCLC/SCLC (1), breast (8), colorectal (3), renal cell (2), others (5)	50^3^	Headache (35), cognitive changes (17), gait issues (7), urinary incontinence (5), seizures (2)	VPS (50/50)	40/50 (80%)	Headache (30), cognitive changes (12), gait issues (5), urinary incontinence (2), seizures (2)	8%; Valve malfunction (1), increased ICP (1), overdrain (1), uncontrolled hydrocephalus (1), infection (0), peritoneal carcinomatosis (0)	1/50 (2%)	3.5 [0–28]b	3 months [2 days–54 months] b
Gonda et al. [23]	Lung (13), melanoma (10), breast (9), renal (3), colon (1)	36^4^	Headache (6); headache and nausea (10); headache, nausea, vomiting (8); headache, lethargy (7); headache, neurological deficit (5)	VPS (36/36)	27/36 (75%)	Headache (3); headache and nausea (8); headache, nausea, vomiting (8); headache, lethargy (7); headache, neurological deficit (1)	19%; wound infection (4), shunt occlusion (2), hygroma (1), peritoneal carcinomatosis (0)	0/36 (0%)	N.R	90 days [47–99 days] for LMD patients only
Jung et al. [24]	NSCLC (37), SCLC (8), breast (14), gastric (4), other N.R (8)	7^5^	N.R	VPS (7/7)	N.R	N.R	N.R	N.R	N.R	5.7 months [95% CI 0.000– 13.173 months]
Yamashiro et al. [13]	Lung adenocarcinoma (4)	4	N.R	LPS (4)	3/4 (75%)	N.R	N.R	0/4 (0%)	N.R	7.5 months [4.5– 15 months]
Murakami et al. [25]	Lung (4), breast (4), ovarian (1), colon (1), nasal rhabdoidsarcoma (1)	11	Headache (10), nausea/vomiting (6), cognitive changes (6), gait issues (4)	LPS (3), VPS (8)	10/11 (91%)	Headache (9), nausea/vomiting (5), cognitive changes (5), gait issues (1)	9%; shunt obstruction requiring revision (1), infection (0), peritoneal seeding (0)	1/11 (9%)	3.9 [3.5–6.3]	3.3 months [2.9– 5.7 months]

*N.R*. not reported, *LPS* lumboperitoneal shunt, *VPS* ventroperiotneal shunt

1 Specific numbers for each symptom not reported

^2^ Range (minimum to maximum survival)

3 Includes 10/50 patients with parenchymal, non-LMD associated hydrocephalus

4 Includes 10/50 patients with parenchymal, non-LMD associated hydrocephalus

5 Study included 71 patients, but only 18 had LMD-associated hydrocephalus, of whom only 7 underwent shunting

Managing LM-associated hydrocephalus in NSCLC is challenging due to the extremely poor prognosis and no consensus on optimal treatment. Lamba et al. (2018) reported that LM-associated hydrocephalus should be managed of a patient-centered and individualized approach [10]. Furthermore, they declared that surgery is necessary for acute ICP reduction, whereas it should not be imposed due to the typically short survival. Hence, the patient’s autonomy should be respected through thorough explanations to both patients and caregivers.

According to our experience, many patients and caregivers declined VP shunt surgery due to poor conditions and fears related to general anesthesia andVP shunt surgery–associated complications, particularly brain hemorrhage around the shunt catheter. Still, VP shunt surgery might be considered the standard procedure for CSF diversion. However, we offered LP shunt surgery under mild sedation to these patients over the years. Most patients agreed to this approach, and our clinical outcome showed similar OS and SFP in both VP and LP shunt surgery groups. For patients who previously missed the opportunity for survival extension and symptom relief due to fear of VP shunt surgery, LP shunt surgery could represent a valuable palliative option. Additionally, it may be a favorable choice for patients with poor consciousness status whose families face less psychological burden.

Although some studies discussed the surgical treatment of LM-associated hydrocephalus, most previous studies focused on patients undergoing VP shunt surgery, with limited research exploring the outcomes after LP shunt surgery. Additionally, the primary cancer types of patients in these studies were often heterogeneous. Zhang et al. (2015) outlined several advantages of LP over VP shunt surgery: (1) it is a simpler and less invasive procedure, (2) it has a shorter operation time and lower infection rate, and (3) it can be performed even with small ventricles [37, 38]. Additionally, Woo et al. (2022) demonstrated the pharmacokinetic advantages of LP shunt surgery when combined with intrathecal chemotherapy via an Ommaya reservoir. Yamashiro et al. (2017) reported 4 cases of LP shunt surgery under local anesthesia for LM-associated hydrocephalus[34]. Su et al. (2021) conducted a comparative analysis of VP (*n* = 33) and LP shunt surgeries (*n* = 7) for LM-associated hydrocephalus, finding no difference in clinical outcomes[31]. Our study, with a larger sample size and a relatively homogeneous NSCLC patient profile, consistently confirmed previous hypotheses and clinical outcomes.

As Lamba et al. suggested, treatment plans should be individualized, considering the patient’s performance status, comorbidities, and prognosis [10]. NSCLC is the most extensively studied solid cancer for molecular targeted therapies. EGFR-TKI agents with strong BBB penetration and effective LM treatment outcomes (such as osimertinib, lazertinib, and zoferitinib) have been recently introduced [2, 9, 20, 28,30]. In our multivariate survival analysis, favorable clinical outcomes were observed in patients with a higher preoperative KPS, those who received postoperative EGFR-TKI treatment, and those without preoperative signs of IICP. These patients could be encouraged to undergo surgery more actively compared to others. Although we could not analyze other genetic backgrounds or treatment options, such as intrathecal therapy, radiotherapy, and systemic chemotherapy, beyond *EGFR* mutations due to the small sample size, future large population studies should include prognostic analyses of these factors. With advancements in cancer treatment, including the emergence of molecular targeted therapies, the importance of precision medicine is increasingly recognized not only for NSCLC but also for breast cancer, malignant melanoma, and SCLC[16, 29, 33, 36]. Consequently, considering prognostic factors for these cancers when establishing treatment strategies is essential in managing LM-associated hydrocephalus. Additionally, further research in this area is needed.

## Limitation

One limitation of this study is the small sample size, which reduces statistical power and limits the ability to detect significant differences. Additionally, among the factors reflecting preoperative status, the VP shunt group had a higher proportion of patients in the poor cancer control group (35% for VP vs. 26.1% for LP), the IICP group (90% for VP vs. 69.6% for LP), the low KPS group (70% for VP vs. 47.8% for LP), and those who did not receive TKI treatment after shunting (70% for VP vs. 66.5% for LP). Although these clinical characteristics did not show statistically significant differences between the two groups, the possibility of selection bias cannot be excluded.

Furthermore, since our institution began performing LP shunt surgery under local anesthesia around 2021, all LM patients—except those with obstructive hydrocephalus—have undergone LP shunt surgery, whereas patients treated prior to this period all received VP shunt surgery. Given the rapid advancements in cancer treatment, this temporal factor may also introduce potential bias.

Therefore, larger, multicenter, and prospective studies are necessary to validate these findings and draw more robust conclusions.

## Conclusion

This study identified several factors potentially influencing SFP and OS in patients with LM-associated hydrocephalus secondary to NSCLC. We found no significant difference in outcomes between VP and LP shunt surgeries regarding OS and SFP. Nevertheless, LP shunt surgery under local anesthesia is recommended to reduce the risks associated with general anesthesia and address patients’ apprehension towardVP shunt surgery. Favorable prognostic factors included a good KPS, effective primary cancer control, absence of IICP at hydrocephalus diagnosis, and active treatments such as EGFR-TKI therapy. These findings underscore the importance of a patient-centered, individualized approach to management prioritizing personal and familial values, ultimately enhancing the quality of life for patients with LM-associated hydrocephalus.

## Data Availability

No datasets were generated or analysed during the current study.
